# Supplementary material for: Short- and long-read metagenomics expand individualized structural variations in gut microbiomes
Source: Nat Commun. 2022 Jun 8;13:3175. doi: 10.1038/s41467-022-30857-9 (PMC9177567; doi:10.1038/s41467-022-30857-9)
Supplement: Supplementary file 1 — Supplementary Information [file 41467_2022_30857_MOESM1_ESM.pdf]

Supplementary Information: Short- and long-read metagenomics expand individualized structural variations in gut microbiomes

J. Wang et al.

This file includes:

Supplementary Figures 1 to 11

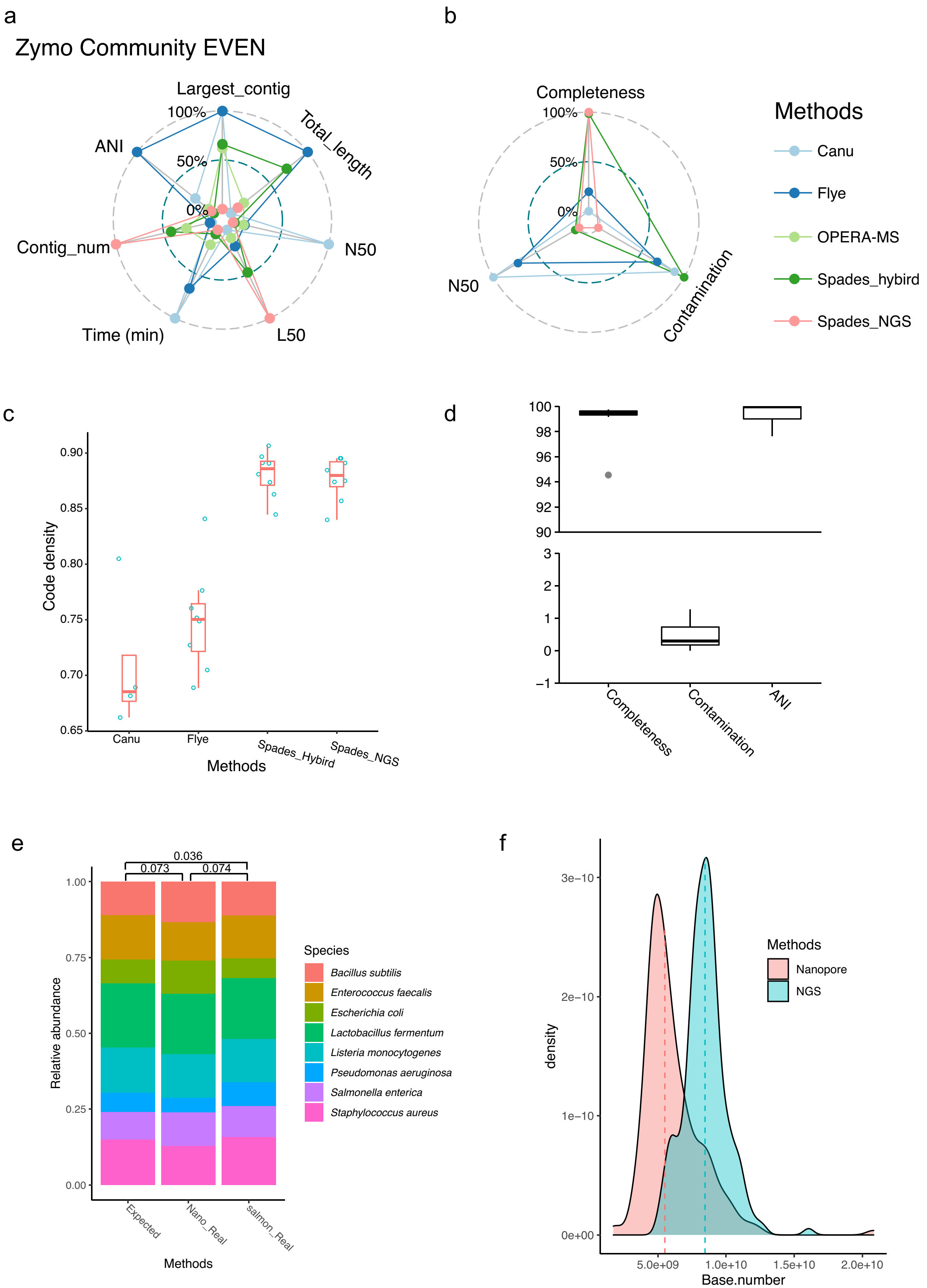
a
Zymo Community EVEN
Largest_contig
Total_length
N50
L50
Time (min)
Contig_num
ANI
100%
50%
0%
b
Completeness
Contamination
N50
Methods
Canu
Flye
OPERA-MS
Spades_hybird
Spades_NGS
c
Code density
Methods
Canu
Flye
Spades_Hybird
Spades_NGS
d
Completeness
Contamination
ANI
e
Relative abundance
Methods
Expected
Nano_Real
salmon_Real
0.036
0.073
0.074
Species
Bacillus subtilis
Enterococcus faecalis
Escherichia coli
Lactobacillus fermentum
Listeria monocytogenes
Pseudomonas aeruginosa
Salmonella enterica
Staphylococcus aureus
f
density
Base.number
Methods
Nanopore
NGS

Supplementary Figure 1. Establishing hybrid assembly pipeline using mock community DNA, related to Figure 1. (a) Evaluation of assembly results in terms of N50, L50 total length, largest contigs, average nucleotide identity (ANI), contig numbers, and running time, between five approaches, including Canu, Flye, OPERA-MS; hybrid assembly using metaSPAdes (Spades-hybrid) and Illumina reads-only assembly (Spades-NGS). (b) Evaluation of binning results in terms of N50, completeness, and contamination, between four approaches that resulted in binned genomes, including Canu, Flye, Spades-hybrid, and Spades-NGS. The approach of OPERA-MS cannot obtain effective metagenome-assembled genomes (MAGs). (c) The coding density of each MAGs obtained from four approaches, including Canu, Flye, Spades-hybrid and Spades-NGS. (d) Summary of the assembly quality of eight bacterial bins from hybrid assembly using metaSPAdes, showing completeness, contamination and ANI. (e) The expected abundance of eight bacterial MAGs (left), versus abundance estimation using ONT reads (middle, Bray-curtis distance 0.073 to expected composition) or Illumina reads (right, Bray-curtis distance 0.036 to expected composition) by Salmon. (f) The distribution of base number of ONT reads (Nanopore) and Illumina reads (NGS) of our two cohorts. Data are presented as box plots (c) (d) with whiskers at the 5th and 95th percentiles, the central line at the 50th percentile, and the ends of the box at the 25th and 75th percentiles.

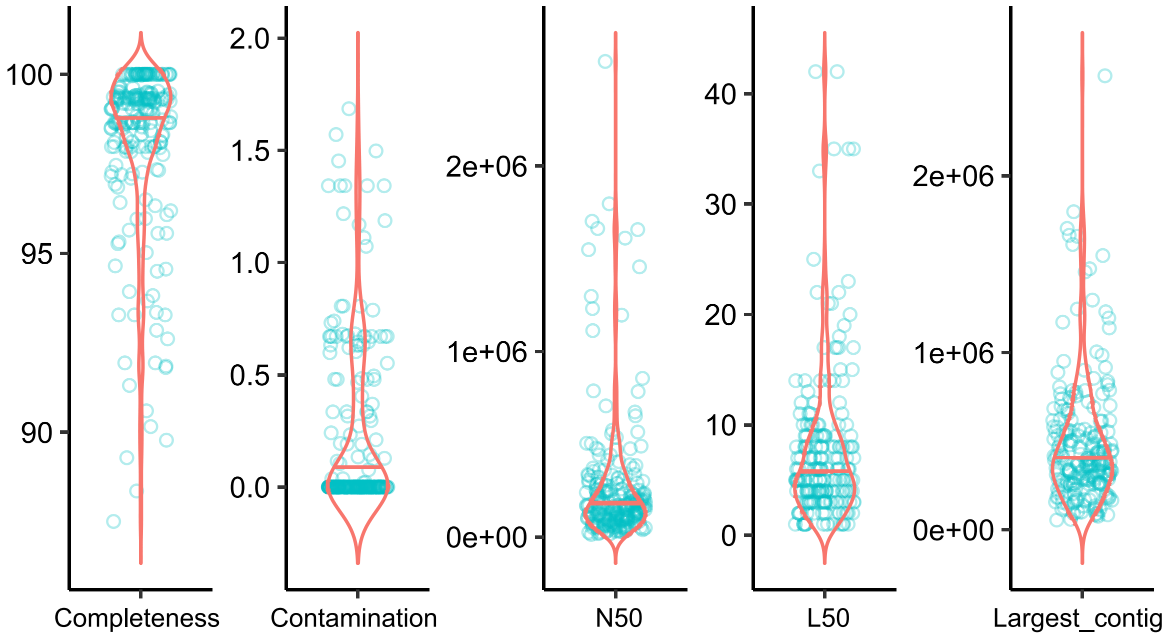

Supplementary Figure 2. The violin plot showing assembly quality of 189 metagenome-assembled genomes (MAGs) (present in > 10 individuals) using for structure variation analysis. The results indicated our MAGs have a good quality (high completeness and low contamination) for the SV detection. Data are presented as violin plot with the central line at the 50th percentile.

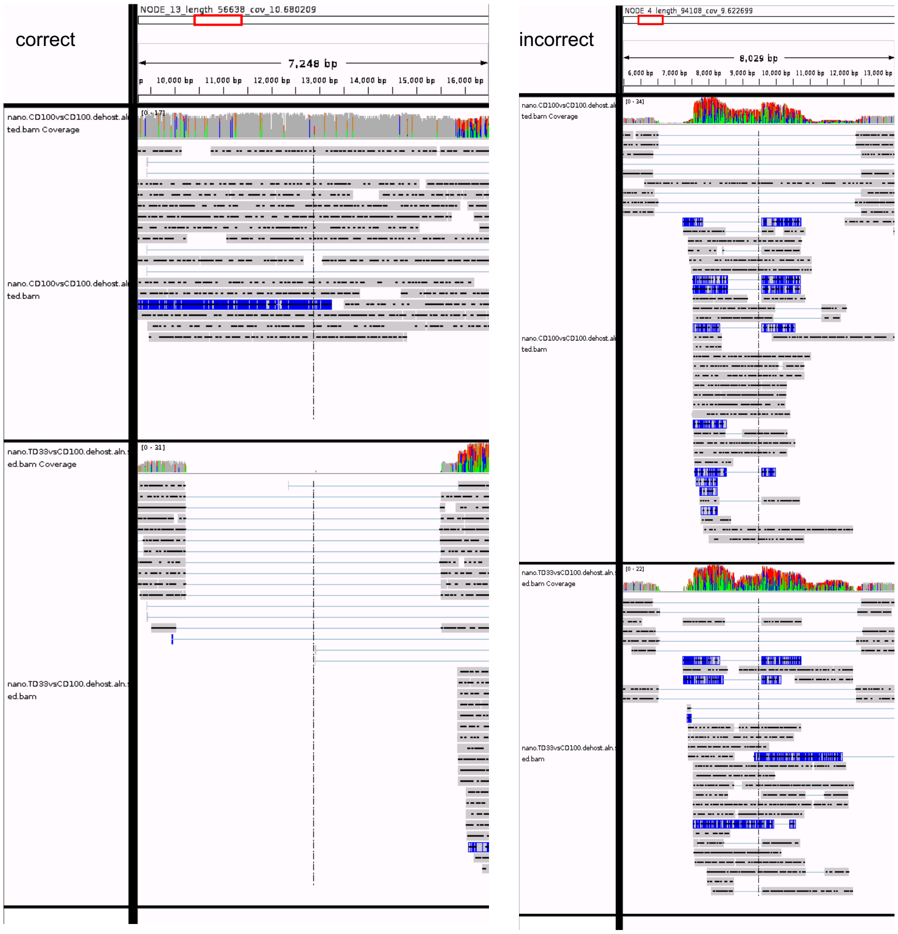

Validation	Numbers	Percent
correct	51	97%
incorrect	2	3%

Supplementary Figure 3. Read-level validation of structural variations in human gut microbiome, related to Figure 2. We randomly chose 53 SVs between a reference metagenome-assembled genome (MAG) and a query MAG, and visualized re-mapping results of ONT reads. The left side shows a correct SV detection, in which the large deletion is present in the lower query MAG, and mapping ONT reads from respective samples against the same reference MAG (upper) supports the presence of large deletion in the query genome. Reversely, large insertions can be also validated using re-mapping against the query genome instead (in this scenario, a large deletion can be found in the reference MAG). The right side shows an incorrect SV discovery, where ONT reads did not fully support the presence of insertions or deletions between MAGs. Among the subset we chose for visual inspection, we estimated that correct SVs accounted for ca. 97%.

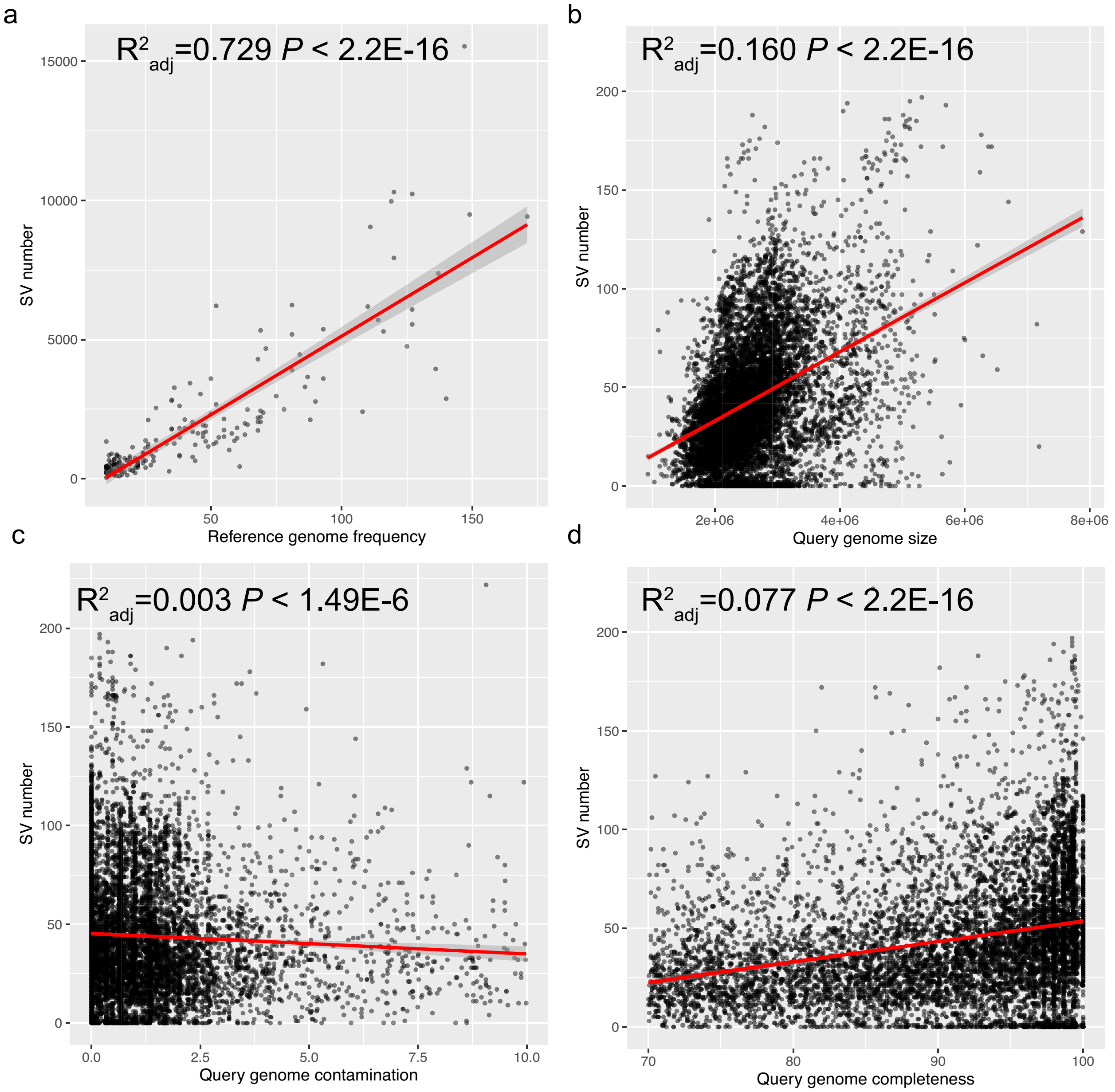

Supplementary Figure 4. The correlation between structural variations and features of metagenome-assembled genomes (MAGs), related to Figure 2. (a) Reference genome frequency (*i.e.* total number of MAGs across the cohort and consequently number of pair-wise comparisons to that of reference), (b) query genome size, (c) query genome contamination and (d) query genome completeness. Simple linear regression model was used to evaluate the correlations between two variables and 95% confidence intervals of fitted line was indicated by shadings. The correlations indicate the genome contamination do not affect the SV number and the necessity to correct for genome size when performing comparisons between different taxonomical groups.

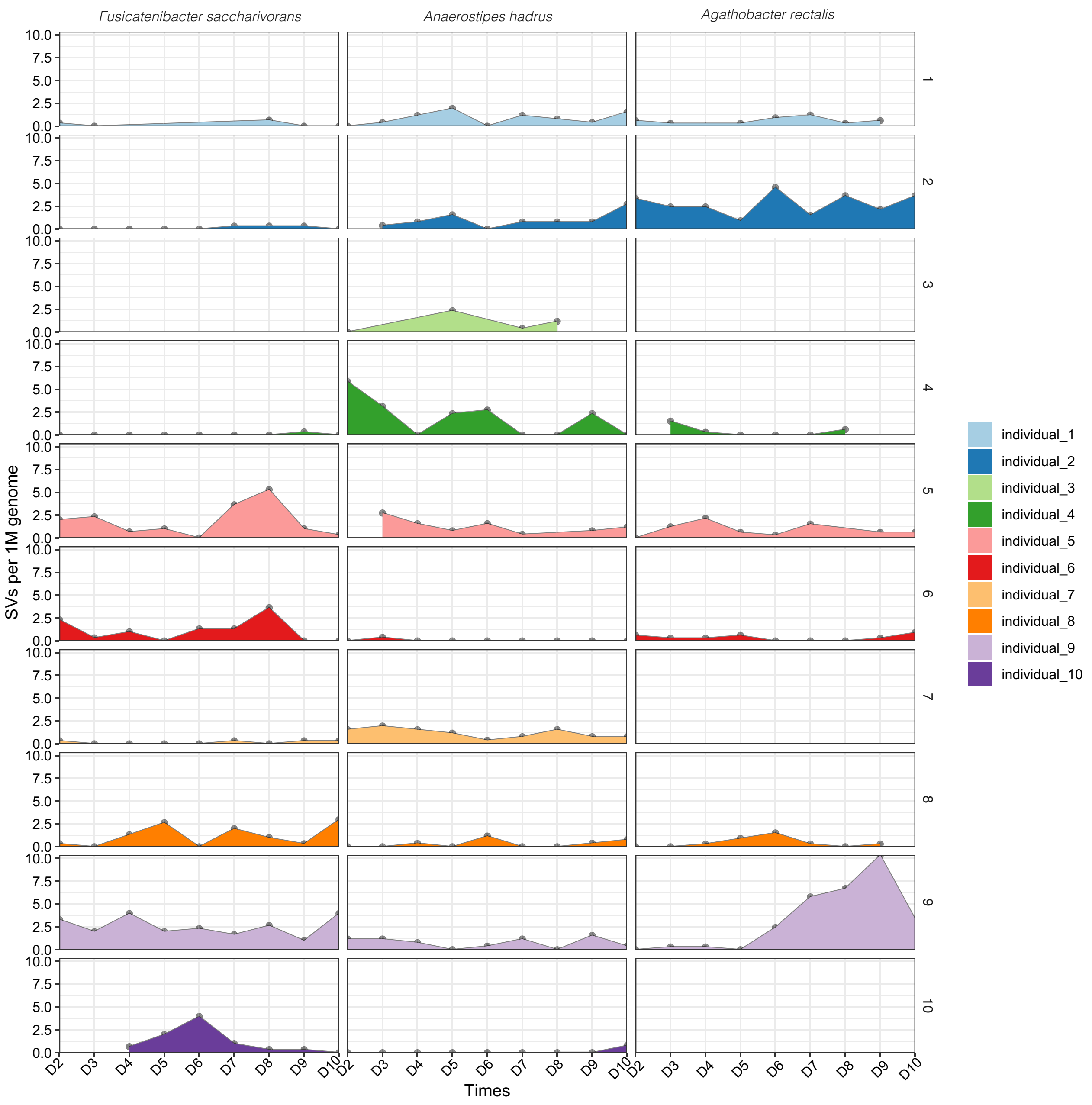

Supplementary Figure 5. Temporal dynamics of structural variations in genome of *Fusicatenibacter saccharivorans*, *Anaerostipes hadrus* and *Agathobacter rectalis* within each individual of our time-series cohort, related to Figure 2. Number of SVs were calculated by referring to the metagenome-assembled genomes (MAGs) of that bacterial species, from the sample collected from the day before, showing very small numbers of SVs (median = 0) within the same individual across 10 days.

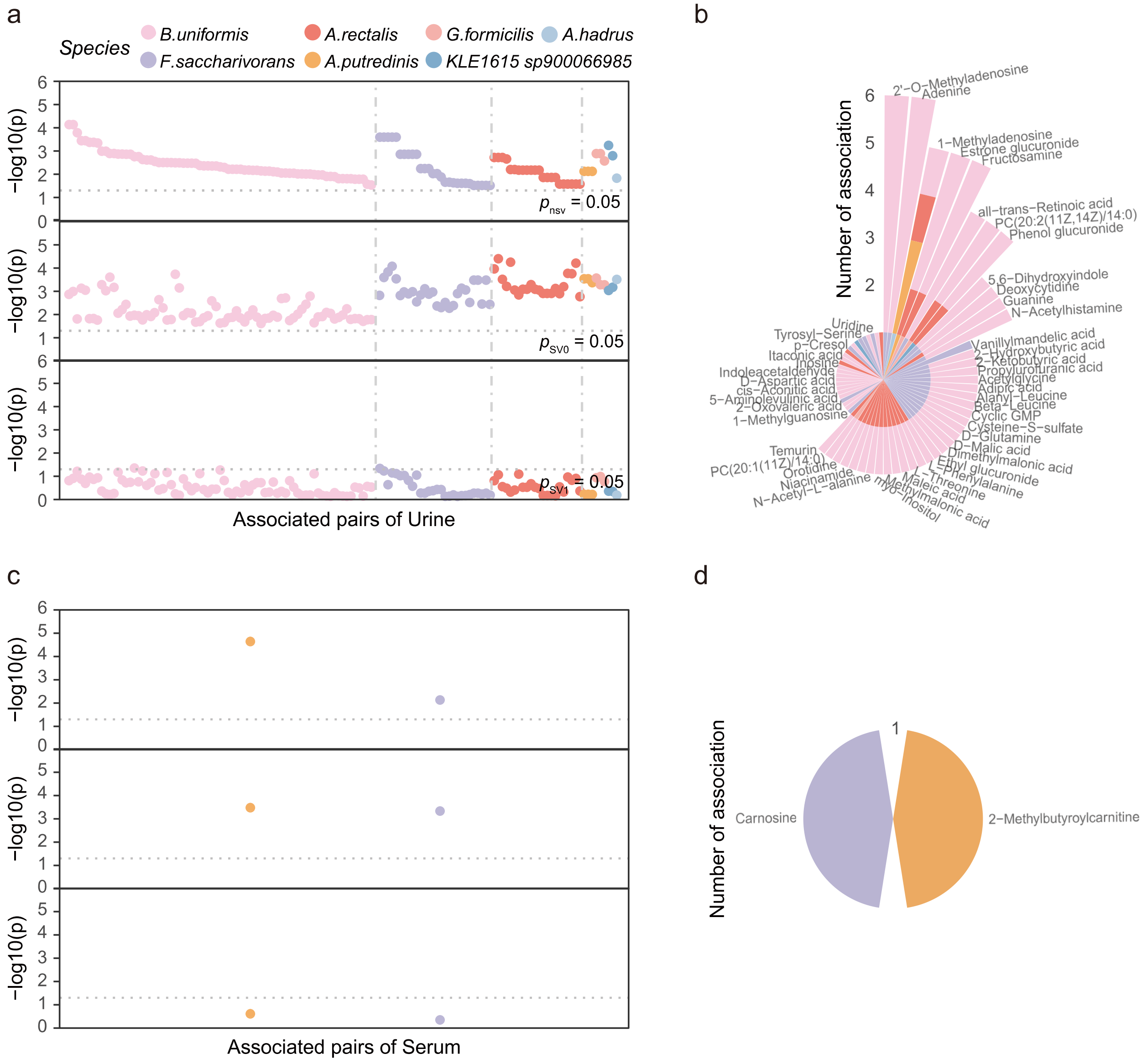

Supplementary Figure 6. SV-affected relevance of gut bacteria and metabolite in human urine and serum, related to Figure 3. (a) Screening for SV-affected correlations between bacteria and urine metabolite. In seven bacteria significantly correlated to 66 metabolites (upper panel, all two-side *p*-values < 0.05, Benjamini-Hochberg FDR < 0.1), the presence of SVs on genes abolished the significant correlations between that subgroup of bacteria with respective metabolites (lower panel, all *p*-values > 0.05). while bacterial subgroup without particular SVs maintained significant correlation (middle panel, all two-side *p*-values < 0.05, Benjamini-Hochberg FDR < 0.2). Colors denote different bacterial species. NSV: all sample; SV0: subgroup without SV in bacterial gene; SV1: subgroup with presence of SV in bacterial gene. (b) Rose

diagram of SV-metabolite correlation pairs in which the presence of SV influences the correlations between bacteria and metabolites (represented by each bar). Colors denote the same bacterial species as in (a), and bar size indicate the number of SVs. (c) Similar to (a), in the two significant correlations between two bacteria and two serum metabolites (upper panel, all p-values <0.05, Benjamini-Hochberg FDR < 0.1), the presence of SVs on genes abolished the significant correlations (lower panel, all p-values > 0.05). However, the absence of particular SVs in bacterial subgroup maintained significant correlation (middle panel, all p-values < 0.05, Benjamini-Hochberg FDR < 0.2). Colors denote the same bacterial species as in (a). (d) Similar to (c), rose diagram detailed the SV-metabolite correlation pairs screened between bacteria and serum metabolites (represented by each bar). Colors denote the same bacterial species as in (a), and bar size indicate the number of SVs.

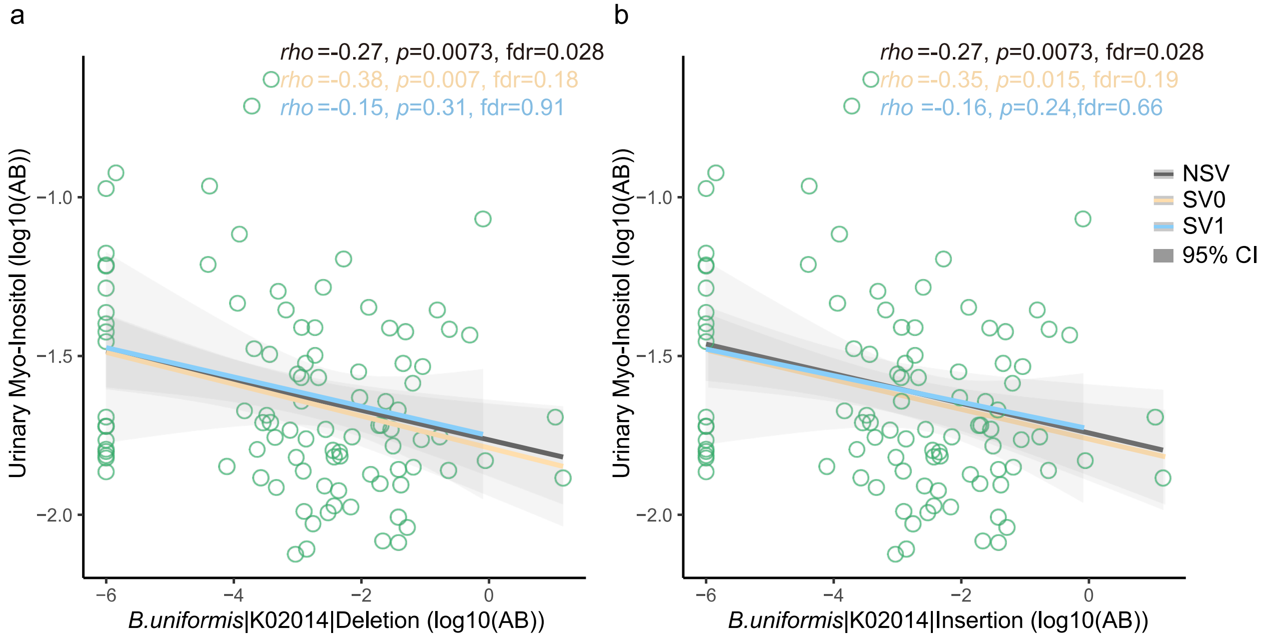

Supplementary Figure 7. SV-affected correlations of *Bacteroides uniformis* and urine Inositol, related to Figure 3. (A) The Urinary myo-inositol (inositol) concentration is significantly correlated with *Bacteroides uniformis* (NSV, n = 100, p = 0.0073, FDR = 0.028), and the subgroup without a deletion on the gene locus (K02014) has similarly significant correlation (SV0, n = 49, *p* = 0.007, FDR = 0.18). However, the presence of deletion abolished this significant correlation (SV1, n = 51, p = 0. 31, FDR = 0.91). (B) Similarly, the subgroup with an insertion on the K02014 also impacted the significant correlation of gut *B. uniformis* and urinary myo-inositol (SV1, n = 62, *p* = 0.24, FDR = 0.66), while the subgroup without insertion on this gene locus maintained significant correlation (SV0, n = 38, *p* = 0.015, FDR = 0.19). The *rho* indicates the coefficient of spearman correlation, and p values were adjusted with Benjamini-Hochberg method. The shadings indicate the 95% confidence intervals (CI). The details of subgroups are available in the Supplementary Table 6. NSV: all sample; SV0: subgroup without SV in bacterial gene; SV1: subgroup with presence of SV in bacterial gene.

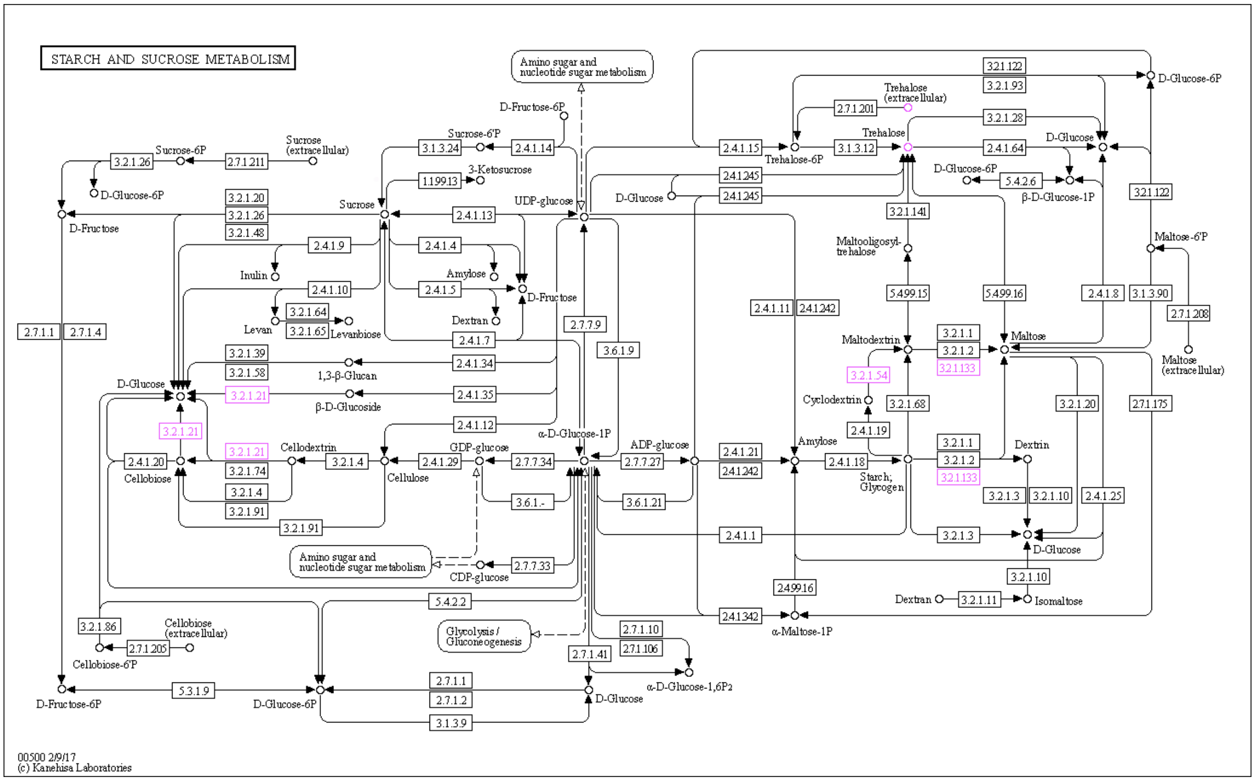

Supplementary Figure 8. The pathway diagram of starch and sucrose metabolism downloaded from KEGG, related to Figure 3. The fecal metabolite (Neotrehalose, the dots in purple) and SV-affected genes (genes of cyclomaltodextrinase whose entry number in KEGG is K01208, and enzyme commission (EC) are 3.2.1.54 and 3.2.1.133 marked purple; gene of beta-glucosidase (K05349), the enzyme EC 3.2.1.21 marked in purple) of gut microbiota were co-mapped to the KEGG pathway, starch and sucrose metabolism. The little hollow dots and rectangles in the diagram indicate compounds and genes involved in pathway, respectively. The ones in purple are our mapping input metabolite ((Neo)Trehalose) and genes (K01208 and K05349).

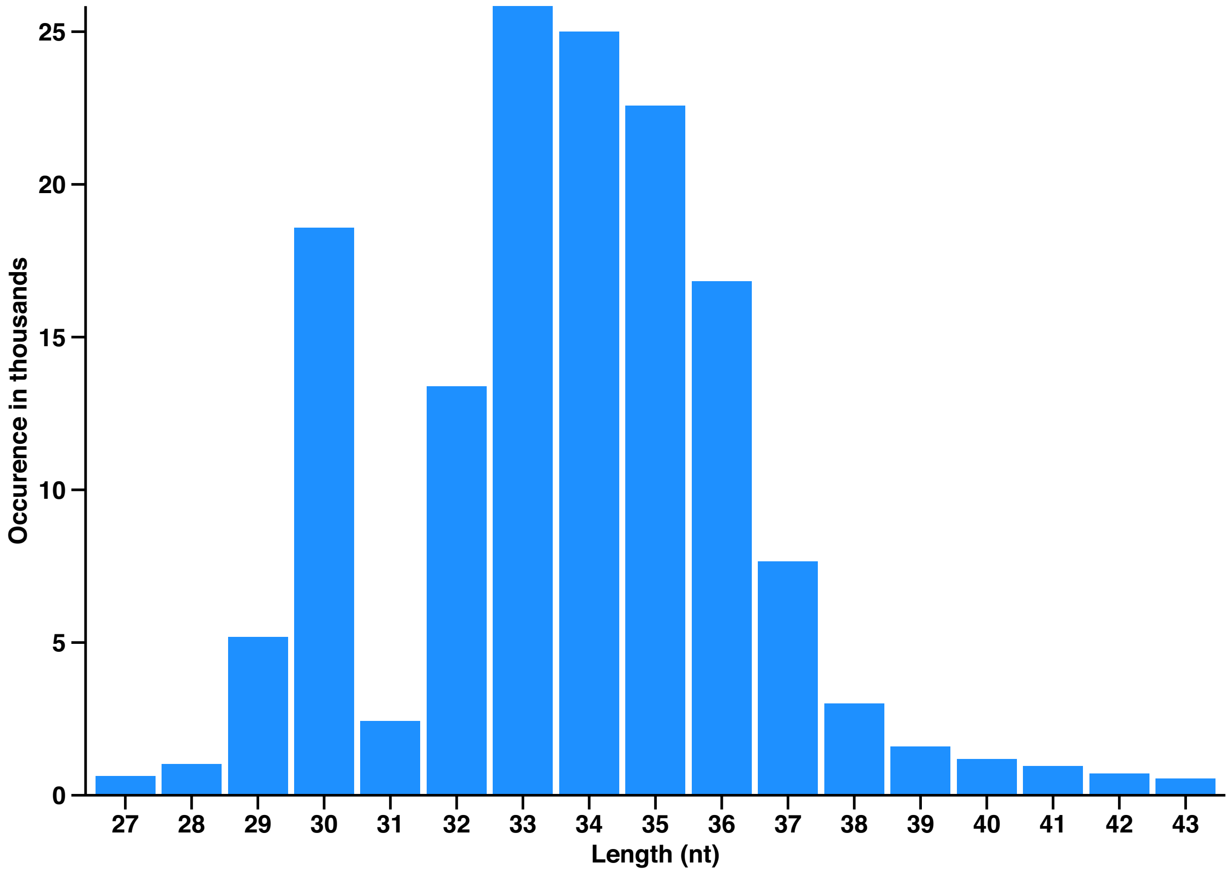

Supplementary Figure 9. Length distribution of CRISPR spacers. The graph shows the number of spacers (Occurrence > 500) with lengths from 27nt to 43nt, with vertical coordinates in thousands.

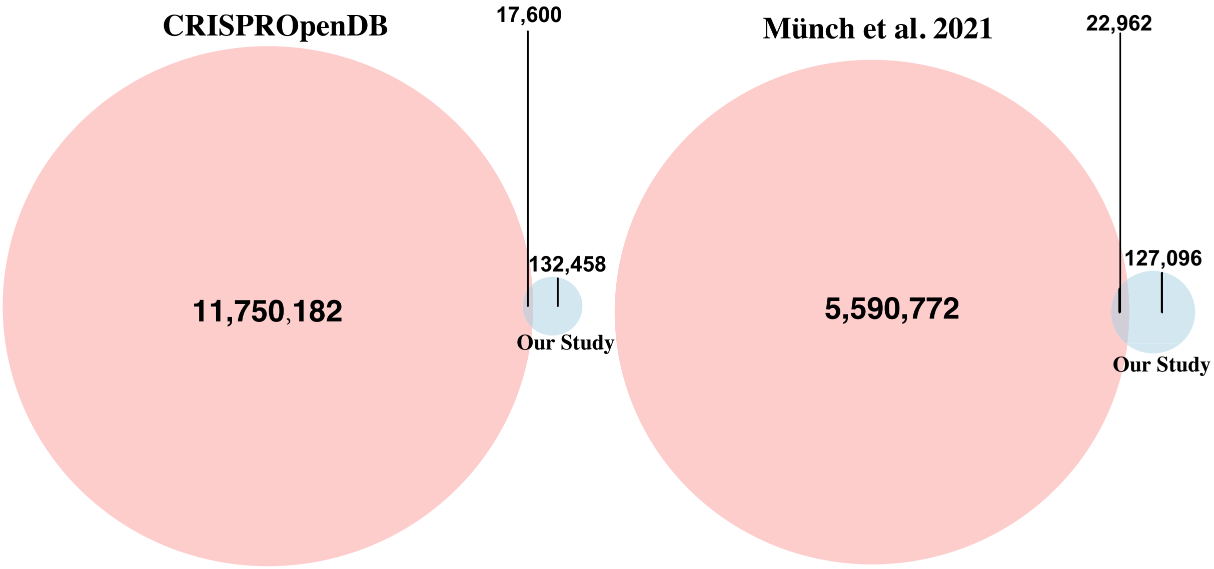

Supplementary Figure 10. Overlap between CRISPR spacers found in our study with published datasets, related to Figure 4. Venn diagram shows the majority of CRISPR spacers are not currently in reported datasets including CRISPROpenDB (left) and spacers discovered in western population gut microbiome by Münch *et al*. 2021 (right). Overlaps were found using blast with a threshold of e-values <1e-5 and < 3 mismatches.

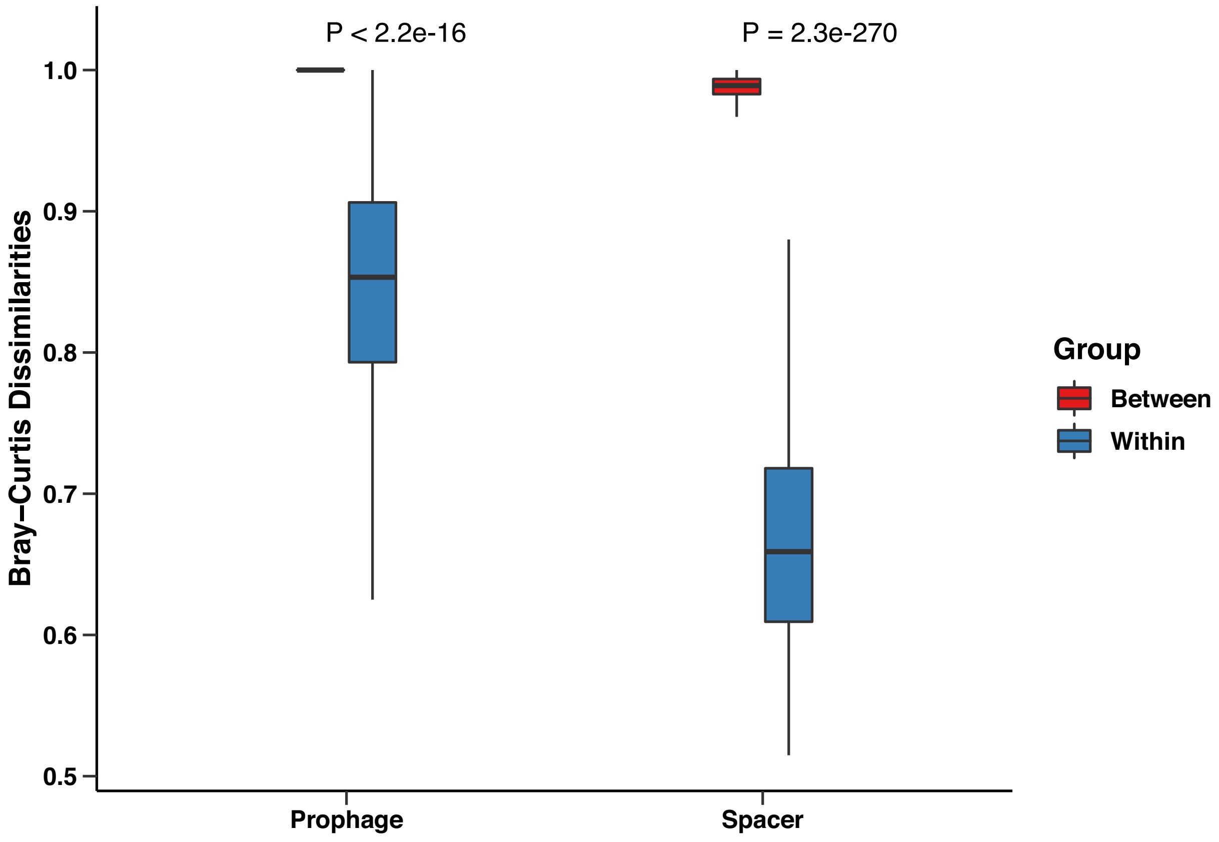

Supplementary Figure 11. Comparison of dissimilarity in prophages and spacers composition between individuals and within individuals, related to Figure 4. The boxplot shows that the inter-individual variation as calculated with Bray-Curtis dissimilarity, from either prophage or spacer composition, was significantly greater than the intra-individual variations by two-sided Wilcoxon test. Prophage (n = 5400, p = 0), Spacer (n = 5400, p = 2.294679e-270). Data are presented as box plots with whiskers at the 5th and 95th percentiles, the central line at the 50th percentile, and the ends of the box at the 25th and 75th percentiles.
