# Supplementary material for: Short- and long-read metagenomics expand individualized structural variations in gut microbiomes
Source: Nat Commun. 2022 Jun 8;13:3175. doi: 10.1038/s41467-022-30857-9 (PMC9177567; doi:10.1038/s41467-022-30857-9)
Supplement: Supplementary file 3 — Description of Additional Supplementary Files [file 41467_2022_30857_MOESM3_ESM.docx]

**Description of Additional Supplementary Files**

File Name: Supplementary Data 1.

Description: The raw results of five assembly methods based on the Zymo Community EVEN data and the binning results based on the assembled contigs.

File Name: Supplementary Data 2.

Description: Summary of clinical characteristics of the cross-sectional cohort (100 healthy individuals).

File Name: Supplementary Data 3

Description: Summary of clinical characteristics of the time-series cohort (10 healthy individuals).

File Name: Supplementary Data 4.

Description: The classification and assembly quality of 189 MAGs (present in > 10 individuals) using for structure variation analysis.

File Name: Supplementary Data 5.

Description: Full result for KEGG pathway enrichment analysis for genes affected by SVs in our cohort. Genes containing breaking points of predicted SVs were annotated by Prokka 1.13 and KEGG Orthology (KO) profiles were annotated by emapper.py 1.0.3. KEGG enrichment analyses were performed using genes of predicted SVs as the foreground genes and all genes of all MAGs as the background. Fisher test was used to caculated the P value and all P-values were adjusted using the Benjamini and Hochberg false discovery rate (FDR) controlling procedure.

File Name: Supplementary Data 6.

Description: Screened gene-metabolites-correlation-pairs affected by SVs in gut bacteria. For each of the SV-metabolite correlations in fecal, urine and serum samples, we listed the bacteria with genes affected by SVs (bacteria|gene|metabolite), and P_NSV, P_SV0, P_SV1 represent the two-side student significances for the whole bacteria species, strains without SVs on this gene locus and with, respectively; similarly Cor_NSV and Cor_SV0 represent the Spearman correlation coefficiencies for the whole bacteria species and strains without SVs respectively. The annotation is provided for each metabolite and ko (POS and NEG are identifiers from metabolome analysis). Spearman correlations were calculated for indicating the relationship between SVs and metabolite.

File Name: Supplementary Data 7.

Description: List of metabolites and SV-affected genes that can be mapped to the same KEGG pathways. In fecal and urine samples, metabolites correlated with SVs and can be mapped to the same KEGG pathways together with the genes affected by SVs are listed, together with annotations for metabolites and genes.

File Name: Supplementary Data 8.

Description: The species-metabolite correlations correcting for age/gender/BMI. For all the 92 species (with SV influencing gene)-metabolite correlations with their metabolites associating with age/bmi/gender, We calculated the correlations again after correcting for age/bmi/gender using linear regression with covariates (Correlation (bacteria|gene|metabolite)), and the Spearman's rho and Pvalue represent Spearman correlation coefficients and two-side significances for bacteria-metabolite pairs, and Beta (Covariates controled) and Pvalue (Covariates controled) indicate the regression coefficients and significances with age/bmi/gender controlled. The annotation is provided for each metabolite and ko (POS and NEG are identifiers from metabolome analysis). Spearman correlations were calculated for indicating the relationship between SVs and metabolite.

File Name: Supplementary Data 9.

Description: Annotations of all identified metabolites in fecal, serum and urine samples . Peak no.: number of identified metabolites; m/z (median): mass-to-charge ratio; Ret. Time (second): Retention time, in minutes (difference in Ret.Time between ES(+) and ES(-) modes was less than XX minutes); Theor. m/z: theoretical monoisotopic mass calculated for the ion (M-H)- , (M+H)+ or (M+FA)-; ES(+) Found m/z: Found m/z- mass detected in the experiment; m/z error (ppm): difference between theoretical and found m/z values in ppm; MSMS fragments: fragments, obtained from the ion (M-H)- , (M+H)+ or (M+FA)-; MS/MS CE (eV): collision energy used for fragmentation optional; Identification level (A; B; C): (A) standard or NMR; (B(i)) confident match based on MS/MS and (B(ii)) confident match using in-silico MS/MS approaches and (B(iii)) partial match based on MS/MS and (C(i)) confident match based on MSn and (C(ii)) confident match using in-silico MSn approaches and (C(iii)) partial match based on MSn; (D) MS only.
