# Supplementary material for: Short- and long-read metagenomics expand individualized structural variations in gut microbiomes
Source: Nat Commun. 2022 Jun 8;13:3175. doi: 10.1038/s41467-022-30857-9 (PMC9177567; doi:10.1038/s41467-022-30857-9)
Supplement: Supplementary file 13 — Reporting Summary [file 41467_2022_30857_MOESM13_ESM.pdf]

nature research

Corresponding author(s): Jun Wang, Moshi Song

Last updated by author(s): May 13, 2022

Reporting Summary

Nature Research wishes to improve the reproducibility of the work that we publish. This form provides structure for consistency and transparency in reporting. For further information on Nature Research policies, see our Editorial Policies and the Editorial Policy Checklist.

Statistics

For all statistical analyses, confirm that the following items are present in the figure legend, table legend, main text, or Methods section.

n/a	Confirmed	
☐	☒	The exact sample size (n) for each experimental group/condition, given as a discrete number and unit of measurement
☐	☒	A statement on whether measurements were taken from distinct samples or whether the same sample was measured repeatedly
☐	☒	The statistical test(s) used AND whether they are one- or two-sided<br>*Only common tests should be described solely by name; describe more complex techniques in the Methods section.*
☒	☐	A description of all covariates tested
☒	☐	A description of any assumptions or corrections, such as tests of normality and adjustment for multiple comparisons
☐	☒	A full description of the statistical parameters including central tendency (e.g. means) or other basic estimates (e.g. regression coefficient) AND variation (e.g. standard deviation) or associated estimates of uncertainty (e.g. confidence intervals)
☐	☒	For null hypothesis testing, the test statistic (e.g. F, t, r) with confidence intervals, effect sizes, degrees of freedom and P value noted<br>*Give P values as exact values whenever suitable.*
☒	☐	For Bayesian analysis, information on the choice of priors and Markov chain Monte Carlo settings
☒	☐	For hierarchical and complex designs, identification of the appropriate level for tests and full reporting of outcomes
☐	☒	Estimates of effect sizes (e.g. Cohen's d, Pearson's r), indicating how they were calculated

Our web collection on statistics for biologists contains articles on many of the points above.

Software and code

Policy information about availability of computer code

Data collection	No specific software was used in data collection.
Data analysis	In this study we used MetaWRAP 1.2, minimap2 v2.17-r941, Canu 1.7, Flye 2.8.1-b1676, OPERA-MS, MetaSPAdes v3.13.0, Quast v.5.0.0, Salmon 0.13.1, Prokka 1.13, MetaSpades v3.13.0, dRep v2.6.2, RNammer -1.2, gtdbtk 1.3.0, MUM&Co v2.4.2, IGV 2.6.2, emapper.py 1.0.3, WGCNA v1.69, R v3.5.2, multtest v2.38.0, ProphageHunter (https://pro-hunter.genomics.cn/index.php/Home/hunter/hunter.html), CD-hit v4.7, PropagAtE, MAFF v7.450, IQTREE2, iTOL v5, CAT, CRSPRDetect v2.4, blast v2.6.0 , CRISPROpenDB, SMRTPortal analysis platform v2.3.0, DEseq2 v 1.26.0, Blastn v2.6.0, MetaBat v2.12.1 and CheckM v1.0.12. Github link is provided for code produced in this work as stated "Code availability: The scripts used for the analysis reported in this study are publicly available at https://github.com/chen318liang/Gut-Metagenome-Pipeline-Based-on-Nanopore-Sequencing.git."

For manuscripts utilizing custom algorithms or software that are central to the research but not yet described in published literature, software must be made available to editors and reviewers. We strongly encourage code deposition in a community repository (e.g. GitHub). See the Nature Research guidelines for submitting code & software for further information.

Data

Policy information about availability of data

All manuscripts must include a data availability statement. This statement should provide the following information, where applicable:

- Accession codes, unique identifiers, or web links for publicly available datasets
- A list of figures that have associated raw data
- A description of any restrictions on data availability

We have now added in the manuscript "Data availability: ONT and Illumina sequencing data generated from this study is deposited in NCBI SRA database with Project ID: PRJNA820119 (https://www.ncbi.nlm.nih.gov/bioproject/PRJNA820119). Source data are provided with this paper."

Field-specific reporting

Please select the one below that is the best fit for your research. If you are not sure, read the appropriate sections before making your selection.

☒ Life sciences ☐ Behavioural & social sciences ☐ Ecological, evolutionary & environmental sciences

For a reference copy of the document with all sections, see nature.com/documents/nr-reporting-summary-flat.pdf

Life sciences study design

All studies must disclose on these points even when the disclosure is negative.

Sample size	We recruited a total of 110 volunteers, including 100 healthy individuals without apparent diseases or infections, and 10 healthy individuals form the time-series cohort. Sample size was not determined as this is a exploratory/descriptive study.
Data exclusions	No data were excluded from the analysis
Replication	Three replicates were performed when necessary, and all replicates have similar performances.
Randomization	Not performed, as this is a exploratory/descriptive study.
Blinding	Not performed, as no grouping or treatment was carried out.

Reporting for specific materials, systems and methods

We require information from authors about some types of materials, experimental systems and methods used in many studies. Here, indicate whether each material, system or method listed is relevant to your study. If you are not sure if a list item applies to your research, read the appropriate section before selecting a response.

Materials & experimental systems

n/a	Involved in the study	
☒	☐	Antibodies
☒	☐	Eukaryotic cell lines
☒	☐	Palaeontology and archaeology
☒	☐	Animals and other organisms
☐	☒	Human research participants
☒	☐	Clinical data
☒	☐	Dual use research of concern

Methods

n/a	Involved in the study	
☒	☐	ChIP-seq
☒	☐	Flow cytometry
☒	☐	MRI-based neuroimaging

Human research participants

Policy information about studies involving human research participants

Population characteristics	We recruited a total of 110 volunteers, including 100 healthy individuals in the city of Beijing, China, who are all local residents, with a 50:50 male:female ratio and age between 25 to 75.
Recruitment	We recruited a total of 110 volunteers that responded to our recruitment calls, and selected individuals based on a common criteria of physical health/absence of diseases and eventually included 100 healthy individuals without apparent diseases or infections, to form the cross-section cohort and each individual provided one fecal, one serum and one urine sample on the same day; and 10 healthy individuals from the same recruitment call form the time-series cohort with 10 consecutive fecal and urine sampling. There are no self-selection bias or other biases that may affect the study.
Ethics oversight	This study is approved by the ethic committee of Institute of Microbiology, Chinese Academy of Science with approval number APIMCAS2021003; all individuals were fully informed and have provided written consent.

Note that full information on the approval of the study protocol must also be provided in the manuscript.
